# Help-Seeking Experiences of Hepatitis B Patients in Transnational Medical Care: The Solution to Health Inequality Is Social Mobility

**DOI:** 10.3390/healthcare7040125

**Published:** 2019-10-31

**Authors:** Chiao-Wen Cheng, Cheng-Min Feng, Chian Sem Chua

**Affiliations:** 1 Department of Transportation & Logistics Management, National ChiaoTung University, Taipei City 100-44, Taiwan; cwcheng.tt97g@nctu.edu.tw (C.-W.C.); cmfeng@mail.nctu.edu.tw (C.-M.F.); 2 Physician, Gastroenterologist & Hepatologist, Western Medicine Division, Hospital Lam Wah Ee 11600, Malaysia; 3 Department of Medicine, Penang Medical College 10450, Malaysia

**Keywords:** health inequality, health policy, hepatitis B, social mobility, socioeconomic status

## Abstract

This paper tracked hepatitis B patients from Medan, Indonesia to Penang, Malaysia under transnational medical care and has an understanding of their medical history and socioeconomic status. The condition of these patients improved as a result of good compliance with medical treatment, including lifestyle adjustment and regular medication. Under the influence of the marketization of healthcare, transnational medical patients in the social structure, based on their economic ability and socioeconomic status, may be expected to experience health inequalities. People with unhealthy medical distribution and weak socioeconomic status are easily prone to diseases due to environmental and social conditions; it is easier for such patients to delay or give up their medical treatment. After continuous tracking and increasing patient exposure to medical knowledge and self-care management opportunities, increasing awareness, screening, care, and treatment, the transmission of hepatitis B can be reduced to enable them to gain upward mobility by their capacities and thus improve their health. Social mobility is deemed the main approach to reduce social inequality. There have been limited medical clinical observations and tracking confirming this theory. This paper, which uses medical observation, confirmed that social mobility is considered as the principal key to reducing inequalities in health.

## 1. Introduction

Hepatitis B virus (HBV) infection is a global and important public health problem Help-Seeking Experiences of Hepatitis B Patients in Transnational Medical Care: The Solution to Health Inequality Is Social Mobility [1]. There are about 248 million hepatitis B carriers in the world. Approximately 786,000 people die each year from liver diseases associated with HBV [2,3]. Hepatitis B carriers are a high-risk group for liver cancer and cirrhosis; moreover, HBV is transmitted to people without hepatitis B antibodies, causing related diseases and increasing the medical burden of society [4,5].

Several countries have implemented transnational medical care plans either to reduce the cost of medical care or to increase the economic benefits for the country. The neoliberal policy of shifting healthcare from a state responsibility to goods, mediated by “free markets”, often leads to the exclusion of the poorest and most vulnerable [6], and allowing free markets to enter the medical industry decreases the state’s responsibility toward ensuring affordable medical care.

In many countries, social inequality causes health inequality and health inequalities resulting from economic inequality are even more serious [7]. The study of social mobility points out that people who move upward in socioeconomic status (SES) through different health mechanisms improve their health, and their health risks are similar to those of higher-level groups [8]. People with lower levels of education generally have lower health status than those who are well educated, and their expected mortality is high [9,10]. Highly educated people have lower unemployment and mortality rates; their physical condition, working conditions, health-related benefits, and pay are superior [11,12]. SES is envisaged as a fundamental cause of health or illness [13]. In chronic disease and health management for the elderly, there are often significant differences in SES, such as personal education, occupation, and income; therefore, personal SES affects health [14,15].

This paper confirms this theory through medical clinical observation of transnational patients with hepatitis B who showed good medical compliance and an improved condition after undergoing health education. In brief, timely diagnosis and treatment of HBV is the key to reducing HBV-related death. We recommend that policymakers take action toward improving social mobility by health education, with the aim of eliminating health inequality.

## 2. Background

Two major issues in Indonesia’s medical care are the lack of medical and human resources. Most of the medical resources are centralized in the capital city of Jakarta. Because the distribution of medical resources is still uneven, the diagnostic level and ability of different levels of medical institutions and staff are still different, resulting in a lower diagnosis rate of chronic hepatitis B patients [16]. Therefore, Indonesian patients often decide to visit the hospitals in neighboring countries, for example, Malaysia, for transnational medical care as a result of word-of-mouth recommendations. Yet, Indonesian patients may experience the economic burden of treatment in Malaysian hospitals because it is difficult for physicians to effectively understand the patients’ medical history, lifestyle, and habits. Besides, it is difficult to follow-up on patients who require long-term follow-up.

Liver cirrhosis remains one of the top-10 causes of death in Indonesia [17]. Every year, 1.4 million people die of viral hepatitis-related cirrhosis and liver cancer worldwide [18]. Estimation of the Global Burden of Disease (GBD) of viral hepatitis has led to increasing international attention [19], and it is one of the major public health concerns around the world [20,21,22], particularly within low–middle-income countries. Nearly one-third of the global population has been infected with HBV, which predisposes the carrier to develop liver cirrhosis, cancer, or liver failure [23]. Hepatitis B is a globally common and severe infectious disease that leads to significant morbidity and mortality [24]. In the southeast region of Asia, about 100 million people have chronic HBV infection. The prevalence of chronic viral hepatitis in this region is 30-times more than that of human immunodeficiency virus (HIV) [25].

Patients with HBV are mainly burdened by the long course of the disease and infection. Even if patients continue to take the antiviral treatment, there will be different outcomes depending on the degree of compliance to take medication or altering their lifestyle [26]. In clinical experience, HBV patients often have insufficient cognitive status, lack of treatment confidence, medication doubts, and unauthorized withdrawal. Therefore, personalized health education is arranged for the patient. The topics include how to use the drug (do not lessen or discontinue the drug by itself), the importance of regular follow-up assessments, quitting alcohol, a balanced diet, and prevention of and knowledge regarding liver disease [27,28]. The main purpose is to enable patients to follow appropriate treatment arrangements, strictly follow the doctor’s advice, not arbitrarily stop taking the drugs, and avoid using drugs that can damage the liver.

Although HBV cannot be completely cured, it is essential to monitor liver condition; adequate medical treatment—when it is necessary—may prevent patients from becoming cirrhotic and developing liver tumors. The best method is to monitor liver condition with abdominal ultrasound and follow-up on liver function regularly [26]. Through regular follow-ups, we established a medical system for hepatitis tracking therapy.

## 3. Cases Materials and Methods

Between October 2016 to January 2017, three patients were treated inadequately in Indonesia for of the symptoms caused by hepatitis B, and they went to Malaysia for medical treatment. After the patients’ condition stabilized, the Patient Consent Form was obtained from the participants and was conducted in accordance with the Declaration of Helsinki. The study did not involve invasive treatment, ethical approval is not applicable, but patients still need to sign a consent form and patient details must be anonymized.

From February 2017 to February 2019, transnational medical Indonesian patients agreed to undergo clinical observation and follow-up using a non-invasive method during their regular follow-up every three months. The social background of these patients is comparable. The annual income is less than the Indonesian GDP of 4,000 US dollars until retirement and their children start working. Their education is secondary-school-level or below; the patients were middle-aged and elderly. After the patients’ condition stabilized, we confirmed the time of each regular follow-up. We first report the patients’ self-rated health in Table A1 [29,30,31] and the open-ended interview knowledge questionnaire of HBV infection, as shown in Table A2, was adapted from Lee et al. [32]. The open-ended interviews explore in greater depth the issues identified to understand: 1. Knowledge levels’ connection to access to HBV care, the source of information. 2. Risk perceptions’ connection to access to HBV care. 3. Healthcare-seeking behaviors. Then, health education was conducted with hepatitis B knowledge and prevention for the patients during the in-depth interviews. After keeping good compliance, these patients’ conditions improved, including symptomatic and quality of life improvements, e.g., lower leg edema, shortness of breath and abdominal distension, through regular follow-up. We sought to corroborate improving the theory of social mobility to solve health inequality through clinical observation and follow-up.

## 4. Results

### 4.1. Social Inequality in Health

The prevalence of economic globalization has made social inequality more pronounced, especially among individuals with a low-income. SES has an important relationship with health. In general, individuals with lower SES are more likely to be exposed to diseases due to their environmental and social conditions [33].

“Before my leg edema, I never went to get a physical examination. When I was sick, I took traditional herbal medicine, When I was sick, I took traditional herb medicine, I didn’t have enough money to go to a doctor. I didn’t know hepatitis B will turn into liver cancer.”

Different SES affect health via different risks. Individuals with high SES have more resources to avoid disease risk, reducing the possibility of disease occurrence, and vice versa [13,33]. For example, medical knowledge and financial deficiencies are a risk for stroke; an individual’s medical knowledge and resources are related to his/her SES. Patients with SES are financially incapable of affording medical checkups and may lack the knowledge and resources to understand the importance of self-healthcare management.

“I am illiterate. My husband died when he was young. The income of this family is based on the clothes I have sewed in the village. When I was in middle age, my neighbor often said that my face was yellow. I always eat traditional herbs; there was no improvement. Until my child graduated from college, the family’s economic situation improved; the child arranged for me to go to have a health screening. The doctor told me that I have hepatitis B infection and jaundice is caused by cirrhosis.”

SES not only affects health but also has an accumulation effect. The health inequalities between different SES groups will gradually expand with age. This interpretation of health inequality is mainly caused by SES that lead to differences in health levels in terms of the work environment, access to medical services, and health risks [34,35,36].

“When I was in high school, I heard hepatitis B from a teacher. After graduation, I became a fisherfolk. I drink at least 5 cans of beer a day for more than 20 years. Until my leg was always swollen and skin itchy, I went to have a physical checkup at a local hospital and noted I have Hepatitis B infection. I have been followed up with the doctor for a long time; my symptoms have been unimproved. I went to the hospital in Malaysia because of abdominal distension. Depending to the doctor’s advice, I realized that hepatitis B needs to be monitored and stop drinking.”

### 4.2. Social Mobility Contributes to a Reduction in Health Inequality

Social mobility is deemed the main approach to reduce social inequality [37]. In a free society, upward social mobility can provide individuals with the ability to engage in various kinds of activities, thus increasing one’s health status. At any time, as long as the individual’s socioeconomic status can be improved, the risk of developing ill-health can be reduced, and upward social mobility should be an effective solution to improve personal health. Therefore, increasing opportunities, education and health knowledge for social mobility enables individuals with low SES to gain upward mobility by their capacities and thus, improve their health.

“It has always been tough while I was young. After retirement, I sold my farmland at the age of 62, my life began to improve. My leg was swollen at aged 65 years old. I went to the hospital for physical checkups, I continued it for 2 years but didn’t get better. I started to have abdominal distension 6 months ago. One day, severe abdominal pain was noted, my family took me to Malaysia for further medical advice. Hepatitis B related liver cirrhosis with liver cancer was diagnosed. After treatment, my leg edema and abdominal distension subsided.”

“After the children started working, the family’s economy was better. My children are engineers and teachers. They said I couldn’t eat herbs, and asked me to go to the doctor. I didn’t know what Hepatitis B is until I went to have a physical checkup.”

The availability of education directly affects what people can achieve and what they succeed in, including the chance for one’s upward social mobility [38]. A study on social mobility indicates that people with upward socioeconomic status learn healthy lifestyles via different mechanisms, such as obtaining the same material level as those of higher-level groups, or the psychological stress they experience decreases, making their health risks similar to those of higher-level groups, thereby promoting their health [8]. The implication that approaches such as promoting health education through community management, increasing patient exposure to medical knowledge, and self-health-care management opportunities could be implemented to narrow the health gaps created by social inequality.

“My liver cancer is treated in Malaysia. After stabilization, the doctor asked me to follow up every three months. Every time I follow up, the doctor will tell me how to take care of myself apart from taking medication, I know that I can’t eat uncooked food, including half-boiled eggs.”

“Before I go to Malaysia for treatment, I don’t know alcohol is not allowed for cirrhotic patients. I must stop drinking; it’s not easy to do so. The doctor encouraged me to stop slowly. The doctor taught me how to record the number of days to drink. This method allows me to cut down my drinking.”

Regardless of current social standing in the overall society, education is the most promising chance of upward social mobility into a better social class and attaining a higher social status [34]. In a society with no mobility, there would be more, rather than less, health inequality. However, increasing social mobility would make a positive contribution to the policy objective of reducing health inequality [8], for example, offering community education and awareness of hepatitis, free screening, vaccination and links to care.

“We stay in remote villages, and people in the village have not received high education. I don’t know there is a hepatitis B vaccine; my children were not given vaccination as well. Until my child accompanied me to Malaysia for treatment, I did know has a vaccine. I asked my children and grandchildren to check to see if they can get a vaccine.”

“My two grandchildren were vaccinated against hepatitis B at birth. My child who is a teacher told me that newborns born in the hospital will be vaccinated if the mother having hepatitis B. However, many people staying in remote villages don’t know the vaccination.”

“Five years ago, my wife passed away; she had a disease similar to mine. I have been to Malaysia for treatment since the doctor provides me the details about hepatitis B. I understand that sexual transmission and vertical transmission from mother and child during delivery are the easiest way to get hepatitis B infection and hepatitis B requires long-term follow-up.”

## 5. Discussion 

The imbalance in the distribution of social resources leads to health inequalities [39]. These unfavorable situations make inequality more serious and, ultimately, make health and mortality inequalities more visible. Eliminating health inequalities has become one of the most important policies of public health departments in various countries [40]. To reduce the incidence and mortality of HBV, it is essential to establish a comprehensive policy of implemented vaccination programs for newborns [24] and ways to actively promote the implemented vaccination in remote villages. 

Education is an integral part of socio-economic status, enabling people to make better use of new information and resources [40]. Disease and treatment knowledge levels are closely related to treatment adherence; therefore, it is important to improve personal knowledge and skills in disease prevention and health promotion [41]. As such, three-quarters of persons infected with HBV do not even know they are infected [42]. Even in high-income countries, poor health is generally associated with poor health knowledge, a greater frequency of medical care, higher healthcare expenditures, or poorer health performance [43,44]. It is necessary to determine how to implement effective raising of awareness, screening, care and treatment and reduce the transmission to minimize the personal and social impact of persons living with hepatitis B. A good educational background facilitates greater access to health care knowledge and the benefits of medical information and technology [40]. With increases in the vaccination rate, the HBV infection rate starts to fall. The most effective way of decreasing HBV infection is vaccination at birth [18,45]. 

Higher socioeconomic status also has advantages in accessibility and use of healthcare resources and services, and a relatively healthier lifestyle [46]. Although the incidence of risk-factor-related diseases has significantly reduced, SES still affects other diseases through different risk factors. Health inequality still exists in all countries. Achieving greater equality in life, death and health issues must break or reduce the link between socio-economic resources and health-promoting preventive measures and treatment by reducing the extent of socio-economic resources and social inequalities [33]. Health is closely linked to the SES of individuals, irrespective of low-, middle- or high-income countries. The policy-makers need to improve social mobility through health education, which is one of the contributing factors to eliminating health inequality.

## 6. Conclusions

Eliminating health inequalities has been one of the most important policies of public health departments in various countries [47]. Through health education, improving social mobility to solve health issues is an urgent health policy requirement in all countries. Therefore, to ensure good national health and well-being, as long as it can improve the social mobility position, it can reduce the risk of ill-health. Upward social mobility should still be an effective solution to improve individual health [48]. While HBV cannot be completely cured, it is important to monitor liver condition. Uptake of testing, vaccination, treatment, and follow-up is essential [45,49]. SES-based health inequalities can be reduced by establishing health education interventions to improve social mobility, provide health checks and knowledge in schools, workplaces, and other community settings, not just through private doctors, to provide health care and the necessary resources [33]. Policies must focus on the social factors of patients’ education to provide effective programs early to achieve national health policy goals and effective response GBD and reduce HBV-related morbidity and mortality.

## Appendix A

**Table A1:** Medical Outcomes Study Questionnaire Short Form 36 Health Survey [29,30].

1. In general, would you say your health is:	
Excellent	1
Very good	2
Good	3
Fair	4
Poor	5
2. Compared to one year ago,	
Much better now than one year ago	1
Somewhat better now than one year ago	2
About the same	3
Somewhat worse now than one year ago	4
Much worse now than one year ago	5

3. The following items are about activities you might do during a typical day. Does your health now limit you in these activities? If so, how much? (Circle One Number on Each Line)

	Yes, Limited a Lot (1)	Yes, Limited a Little (2)	No, Not limited at All (3)
a. Vigorous activities, such as running, lifting heavy objects, participating in strenuous sports	1	2	3
b. Moderate activities, such as moving a table, pushing a vacuum cleaner, bowling, or playing golf	1	2	3
c. Lifting or carrying groceries	1	2	3
d. Climbing several flights of stairs	1	2	3
e. Climbing one flight of stairs	1	2	3
f. Bending, kneeling, or stooping	1	2	3
g. Walking more than a mile	1	2	3
h. Walking several blocks	1	2	3
i. Walking one block	1	2	3
j. Bathing or dressing yourself	1	2	3

4. During the past 4 weeks, have you had any of the following problems with your work or other regular daily activities as a result of your physical health? (Circle One Number on Each Line)

	Yes (1)	No (2)
a. Cut down the amount of time you spent on work or other activities	1	2
b. Accomplished less than you would like	1	2
c. Were limited in the kind of work or other activities	1	2
d. Had difficulty performing the work or other activities (for example, it took extra effort)	1	2

5. During the past 4 weeks, have you had any of the following problems with your work or other regular daily activities as a result of any emotional problems (such as feeling depressed or anxious)?(Circle One Number on Each Line)

	Yes	No
a. Cut down the amount of time you spent on work or other activities	1	2
b. Accomplished less than you would like	1	2
c. Didn’t do work or other activities as carefully as usual	1	2
6. During the past 4 weeks, to what extent has your physical health or emotional problems interfered with your normal social activities with family, friends, neighbors, or groups?		
Not at all	1	
Slightly	2	
Moderately	3	
Quite a bit	4	
Extremely	5	
7. How much bodily pain have you had during the past 4 weeks?		
None	1	
Very mild	2	
Mild	3	
Moderate	4	
Severe	5	
Very severe	6	
8. During the past 4 weeks, how much did pain interfere with your normal work (including both work outside the home and housework)?		
Not at all	1	
A little bit	2	
Moderately	3	
Quite a bit	4	
Extremely	5	

These questions are about how you feel and how things have been with you during the past 4 weeks. For each question, please give the one answer that comes closest to the way you have been feeling. (Circle One Number on Each Line)

9. How much of the time during the past 4 weeks

	All of the Time	Most of the Time	A Good Bit of the Time	Some of the Time	A Little of the Time	None of the Time
a. Did you feel full of pep?	1	2	3	4	5	6
b. Have you been a very nervous person?	1	2	3	4	5	6
c. Have you felt so down in the dumps that nothing could cheer you up?	1	2	3	4	5	6
d. Have you felt calm and peaceful?	1	2	3	4	5	6
e. Did you have a lot of energy?	1	2	3	4	5	6
f. Have you felt downhearted and blue?	1	2	3	4	5	6
g. Did you feel worn out?	1	2	3	4	5	6
h. Have you been a happy person?	1	2	3	4	5	6
i. Did you feel tired?	1	2	3	4	5	6

10. During the past 4 weeks, how much of the time has your physical health or emotional problems interfered with your social activities (like visiting with friends, relatives, etc.)? (Circle One Number)	
All of the time	1
Most of the time	2
Some of the time	3
A little of the time	4
None of the time	5

11. How TRUE or FALSE is each of the following statements for you. (Circle One Number on Each Line)

	Definitely True	Mostly True	Don’t Know	Mostly False	Definitely False
a. I seem to get sick a little easier than other people	1	2	3	4	5
b. I am as healthy as anybody I know	1	2	3	4	5
c. I expect my health to get worse	1	2	3	4	5
d. My health is excellent	1	2	3	4	5

**Table A2:** The open-ended interview with key informants.

Questions
1. What do you think you need to know about hepatitis B?
2. What do you think you need to know about the healthcare services for hepatitis B?
3. Which sources of information do you use most to inform your health seeking?
4. How did you have your test/vaccination/check-up/treatment?
5. What made you have the test/vaccination/check-up/treatment?
6. How do you look after your health in general?
7. Do you have your preferred ways of keeping healthy? If yes, what are they? Why?
8. Under what circumstances will you seek help from health professionals?
9. Do you ever discuss with your doctor regarding your preference of treatment? Why yes, why not?
10. What do you do after seeing a doctor? Will you take the medicine? Why yes, why not?
